# Effect of Radiation up to 30 MGy on Mechanical and Dielectric Properties of Polymers for Superconducting and Resistive Magnets

**DOI:** 10.3390/polym18040448

**Published:** 2026-02-10

**Authors:** Christian Scheuerlein, Filip Louka, Pavan Chaganti, Roland Piccin

**Affiliations:** European Organization for Nuclear Research (CERN), Esplanade des Particules 1, 1211 Geneva, Switzerland

**Keywords:** irradiation, epoxy resin, 3D printing, PEEK, PPS, PEI, polyurethane, casting, elastomer, superconducting magnet, accelerator, fusion, break down voltage, mechanical strength, shear strength, three-point bending, cross-linking, chain scission

## Abstract

The effect of ionising irradiation up to 30 MGy on the mechanical and dielectric properties of different polymers for potential use in particle accelerators and detectors was compared in this study. The materials studied include the high-performance polymers PEEK, PPS and PEI; pure anhydride- and amine-based epoxy resin systems for coil impregnation and adhesive bonding; glass fibre epoxy composites; and FDM, SLA and SLS 3D-printed materials and polyurethanes. Gamma irradiation was applied in ambient air at an approximate dose rate of 2 kGy/h. Dose-dependent radiation damage was monitored by three-point bending tests, Shore A hardness, tensile stress–strain measurements and breakdown voltage tests in liquid nitrogen. Radiation hardness was rated according to two criteria: the dose at which the initial mechanical strength is halved and the dose at which the mechanical strength is reduced below a certain threshold value. The degradation of the breakdown voltage was preceded by the degradation of mechanical properties.

## 1. Introduction

To qualify polymers for use in particle accelerators, detectors, and fusion devices, their resistance to ionising irradiation needs to be characterised [1,2,3,4,5,6,7,8]. Nb_3_Sn superconductor coils will be used in the magnets of future accelerators like those of the High Luminosity Large Hadron Collider (HL-LHC) [9,10,11], the Future Circular Collider (FCC) [12,13] or the Muon Collider [14]. Nb_3_Sn coils need to be impregnated with a resin [15] to prevent conductor damage under high mechanical stresses [16]. In particle accelerators and detectors, polymers are also needed as dielectric spacers and adhesives [17], as sealants, for alignment tools [18] and for shimming. For the HL-LHC final focus magnets, the dose absorbed by organic insulation materials will be limited to 30 MGy [19,20,21].

In the present study, we characterised radiation damage in a wide range of organic materials for potential use in particle accelerators and detectors, as well as in fusion devices. The materials irradiated include pure epoxy resin systems for impregnation and for adhesive bonding; glass-fibre-reinforced epoxy composites; 3D-printed materials produced by Fused Deposition Modelling (FDM), Stereolithography (SLA) and Selective Laser Sintering (SLS); and injection-moulded and extruded high-performance polymers.

Irradiation up to 30 MGy was applied with a gamma ray source in ambient air with a dose resolution between 0.5 MGy and 5 MGy. The irradiation-induced ageing of polymers that are rigid at room temperature (RT) was assessed by three-point bending tests. For polymers whose glass transition temperature is above RT, radiation effects were monitored by Shore A hardness measurements and tensile tests. For many polymers, an initially fast decrease in mechanical properties is followed by a plateau during which the mechanical properties are only slightly changed with increasing dose. The criteria that are used to compare the dose limits of the materials tested include a 50% reduction in bending stress at fracture (*σ*_max_) and the dose at which the bending stress is decreased below a threshold value of *σ*_max_ = 50 MPa.

The most important function of polymers in accelerator magnets is the dielectric insulation of live parts. To verify the effect of ionising irradiation on the insulating properties, mechanical test results were compared with breakdown voltage tests after identical irradiations of the same materials. Breakdown voltage tests were performed in liquid nitrogen to represent conditions in superconducting magnets immersed in liquid helium. It was shown that a reduction in breakdown voltage is preceded by a drastic reduction in mechanical strength.

## 2. Materials and Methods

### 2.1. The Samples

Pure epoxy resin samples were produced as 4 mm thick plates by vacuum impregnation at the CERN polymer laboratory. Polyurethane (PUR) plates were either received in cast form or were produced by vacuum impregnation. All samples of a given material were cut from the same plate by a water jet. Glass-fibre-reinforced composite samples were either produced by vacuum impregnation or were machined out of magnet components. Final test sample geometries were produced by 3D printing and by injection moulding.

### 2.2. Iradiation

Gamma irradiation with a ^60^Co source was performed at the Gammatec facility at the Marcoule site of the company Synergy Health Marseille SAS, France (a Steris company), with a dose rate of about 2 kGy/h, in ambient air at a temperature of 20–25 °C. During irradiation, the sample holders were continuously rotated for better dose homogeneity. Unless explicitly stated, the uncertainty of the dosimetry results is ±5%. More details about the irradiation procedures can be found in [22].

### 2.3. Tensile Stress–Strain Measurements

Uniaxial tensile stress–strain measurements based on ISO 527 [23] were performed using DIN 50125-E 3 × 8 × 30 samples, with a constant crosshead speed of 100 mm/min. Strain was measured with an extensometer with a gauge length of 10 mm. The engineering stress was calculated from the cross-section of the unloaded samples.

### 2.4. Three-Point Bending Tests

To reduce the sample volumeto be irradiated, three-point bending tests were performed in short beam configuration with 40 mm long samples according to ISO 14130:1997 [24], instead of flexural tests according to ISO 178 [25], which require 80 mm long samples. Samples with 10 mm width and with a nominal thickness of 4 mm were tested using Ø = 4 mm loading supports and a Ø = 10 mm bending die. The span length was 5× the nominal sample thickness, and the crosshead speed was 1 mm/min. The flexural stress and flexural strain of the samples without fibre reinforcement were calculated according to Equations (1) and (2) from load (*F*), specimen width (*b*), specimen thickness (*h*), support span (*L*), and displacement (*s*):

(1) $$Flexural\;stress\;\left(MPa\right):\;\sigma \;=\;\;\frac{3\;\times \;F\;\times \;L}{\begin{array}{c} 2\;\times \;b\;\times \;h^{2} \end{array}}$$

(2) $$Flexural\;strain\;\left(\%\right):\;\varepsilon =\frac{6\times s\times h}{L^{2}}\times 100\%$$

It is estimated that the three-point bending strength determined in the short beam configuration typically exceeds that determined by standardised flexural tests by 10–20%. This difference was derived empirically by comparing the three-point bending stress results obtained according to ISO 178 (flexural strength) and according to ISO 14130:1997 (short beam strength) for identical materials.

The shear strength (*τ*) of the matrix material in the glass-fibre-reinforced composite samples was calculated from maximum load (*F*), specimen width (*b*) and specimen thickness (*h*):

(3) $$Shear\;strength\;\left(MPa\right):\;\tau \;=\;\;\frac{3\;\times \;F}{\begin{array}{c} 4\;\times \;b\;\times \;h \end{array}}$$

To account for the anisotropic properties of composite materials, in the following sections, we refer to τ as apparent shear strength.

### 2.5. Shore A Hardness

Shore A hardness values were derived using a Zwick durometer. The penetration of the indenter into the 4 mm thick specimen was measured under a load of 1 kg after a dwell time of 3 s. The results presented are the arithmetic mean of 5 measurements.

### 2.6. Breakdown Voltage Measurements

Electrical breakdown measurements were carried out in accordance with IEC 60243-1 [26]. A FUG DC high-voltage power supply (HCP 350-65000) was used for the DC breakdown voltage measurements using two flat 21 mm diameter brass electrodes. The power supply was controlled via a LabVIEW interface. Positive polarity voltage was applied in a stepwise manner with a ramp rate of 500 V/s until electrical breakdown occurred, with the current limit set to 500 µA. After each experiment, the electrode surfaces were cleaned. Results are the average of at least 5 tests per material.

Some of the polymer samples tested may contain macroscopic holes and cracks. The breakdown voltage measured for such samples is determined either by the dielectric properties of the medium that fills these holes or by those of the dense polymer material, whichever breakdown voltage is lower.

The breakdown voltage values reported here were determined for samples immersed in liquid nitrogen (LN2). Like liquid helium, LN2 is a medium with good dielectric properties. Unlike LN2, air is a medium with poor dielectric properties. The breakdown voltage of a 0.5 mm air gap is only about 1.5 kV.

## 3. Results

Below, the irradiation-induced changes in the mechanical and dielectric properties of different polymer materials for possible application in superconducting magnets are compared.

### 3.1. Pure Epoxy Resins for Coil Impregnation

**Figure 1:**
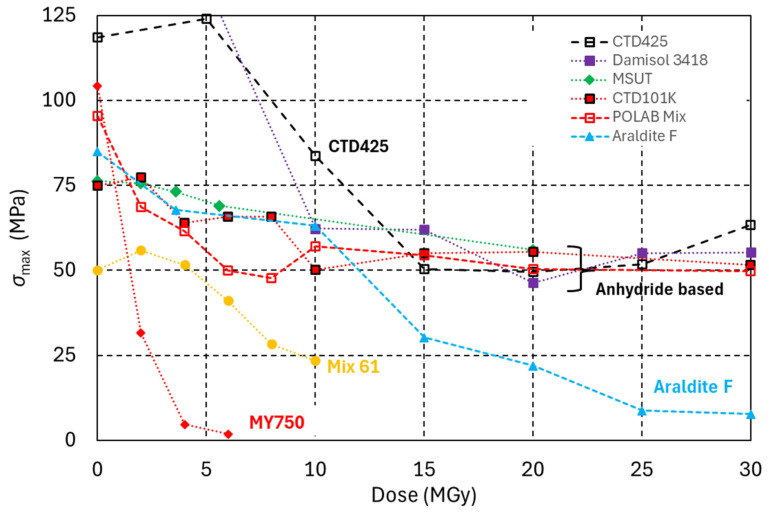
Maximum three-point bending stress (*σ*_max_) of different epoxy resin systems as a function of radiation dose.

In Figure 1, the dose-dependent maximum three-point bending stress (*σ*_max_) evolution of five anhydride-cured DGEBA epoxy systems is compared with that of two epoxy systems with amine-based hardeners and of one epoxy cyanate ester blend.

The anhydride hardener-based epoxy resin systems CTD101K [27], POLAB Mix, Araldite F [28], and MSUT [29] and the amine-based systems MY750 [30] and Mix 61 [31,32] are identical to those described in detail in references [33,34]. Damisol 3418 is a one-component class H high-voltage solvent-free DGEBA epoxy–anhydride hardener system [35]. The curing cycle comprised two isothermal plateaus at 120 °C (4 h) and at 160 °C (8 h).

CTD 425 is a two-part system consisting of an epoxy resin and a cyanate ester catalyst. Sixty parts epoxy resin and forty parts cyanate ester were mixed and degassed at 50 °C [36]. The curing temperature cycle comprised two isothermal plateaus at 100 °C (22 h) and at 150 °C (24 h). During the curing process of cyanate esters, a very stable triazine ring network is formed. Due to its comparatively higher irradiation resistance, CTD 425 is used for the impregnation of the ITER toroidal field coils [37].

Epoxy systems with anhydride-based hardeners are much more radiation-hard than those with amine-based hardeners. After an initial strong degradation of mechanical properties in the dose range 5 to 15 MGy, a plateau is reached, and higher doses do not cause a strong *σ*_max_ degradation of these materials up to at least 30 MGy.

POLAB Mix is a flexibilised four-component epoxy system which has improved mechanical properties with respect to CTD101K, which is the baseline impregnation resin for the HL-LHC Nb_3_Sn final focus magnets [35]. POLAB Mix consists of the three components of the CTD101K system and the Araldite DY040 flexibiliser as a fourth component. As can be seen in Figure 1, the addition of the flexibiliser does not alter the radiation hardness, at least up to 30 MGy.

The Araldite F epoxy system with the anhydride hardener blend Araldite HY905 is less radiation-hard than epoxy systems with single anhydride compound hardeners like HY906. The epoxy cyanate ester blend (CTD425) exhibits comparatively smaller degradation after 10 MGy.

### 3.2. Fibre-Reinforced Epoxy Composites

**Figure 2:**
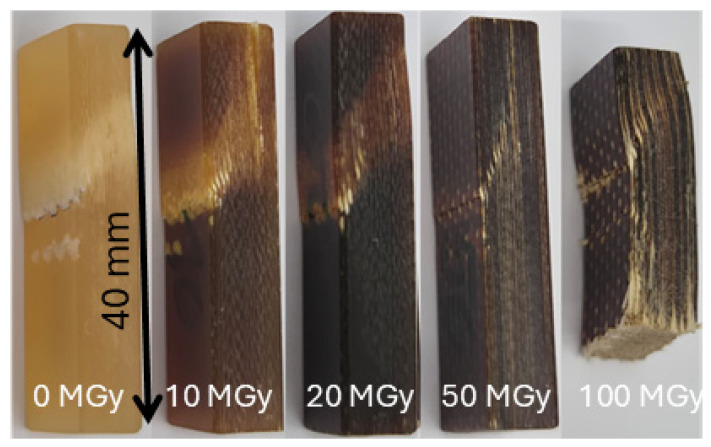
Photographs of the CTD101K-S2 glass [0/90] samples after fracture in three-point bending in short beam configuration.

The apparent shear strength of the following glass-fibre-reinforced epoxy composites (GFRE) was measured before and after irradiation:

- ISOPREG 2704 is an E-glass-fibre-reinforced epoxy prepreg from Isovolta that is used in the LHC corrector magnets MCBC/MCBY [38]. The composite was loaded in the [0,90] fibre orientation.
- “GFRE end spacer lab 1” and “GFRE end spacer lab 2” samples were extracted in random fibre orientation from glass-fibre-reinforced epoxy laminate of end-spacers of the LHC final focus magnets MQXA [39].
- The CTD101K-S2 glass composite is used in the HL-LHC final focus magnets MQXFB. The samples have a S2 glass volume fraction of *V*_f_ = 33% and were loaded in the [0/90] fibre orientation. Photographs of the CTD101K-S2 glass [0/90] samples after fracture are presented in Figure 2. The main failure mode in the short beam configuration is diagonal shear failure [40].

**Figure 3:**
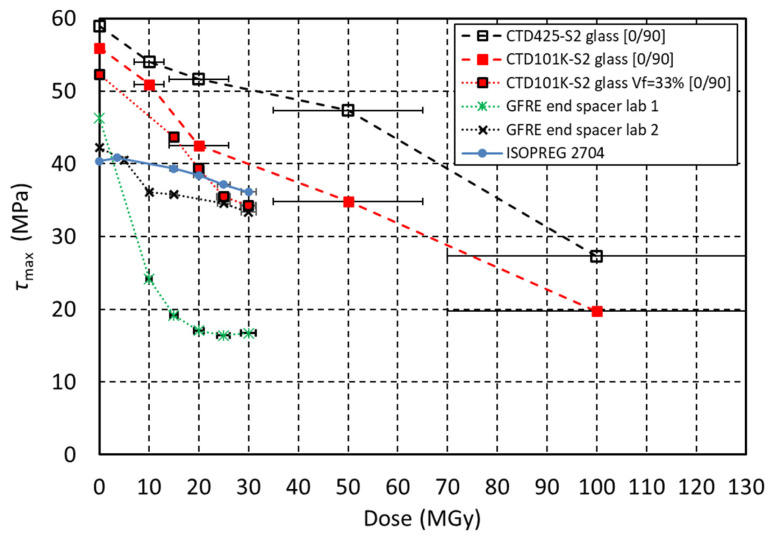
Maximum short beam stress (*τ*_max_) of the epoxy resin matrix in different glass-fibre-reinforced composites as a function of radiation dose. Error bars represent dosimetry uncertainty of ±5% and ±30 MGy.

In Figure 3, the maximum apparent shear strength of the epoxy matrix of the different GFRE is plotted as a function of the absorbed dose.

The CTD101K composite with *V*_f_ = 33 vol.% S2 glass fibre content, ISOPREG 2704 and GFRE from lab 2 are only slightly degraded after a dose of 30 MGy. In contrast, *τ*_max_ of the GFRE used for the MQXA end-spacers from lab 1 is halved after 10 MGy, indicating that the epoxy matrix of this composite is less radiation hard.

For comparison, the apparent shear strength values measured on S2 glass-reinforced composites with CTD101K and CTD425 matrix are shown as well. The composite samples were previously irradiated with a gamma source with a comparatively higher dose rate in the order of 10 kGy/h up to doses in the order of 100 MGy. The error bars of these data points represent a dosimetry uncertainty of ±30% [41]. It can be roughly estimated that after a dose in the order of 100 MGy absorbed in air, the most radiation-hard epoxy resins and the CTD425 cyanate ester epoxy blend may maintain a shear strength of roughly 20 MPa and 30 MPa, respectively.

### 3.3. Epoxy Adhesives

Below, the radiation hardness of four epoxy systems with amine-based hardeners that can be used as adhesives for cryogenic applications is compared:

- Araldite 2011 is a two-component RT-curing epoxy adhesive with an amine-based hardener [17].
- Eccobond 286, also known as Loctite Ablestik 286, is a two-component RT-curing epoxy adhesive with an amine-based hardener [42].
- Stycast 2850FT [43] is a charged epoxy resin for cryogenic applications [44]. It was studied with the 23LV polypropylene glycol diamine, 3,3’-Oxybis(Ethyleneoxy)Bis(Propylamine) hardener. Because of its particle charge, the epoxy system has comparatively high thermal conductivity at a cryogenic temperature [45], and its coefficient of thermal expansion (CTE) of 44 × 10^−6^ K^−1^ is about 25% lower than the CTE of pure epoxy resins [34].
- The Stycast E4215+HD3561 system is used, for instance, for potting of high-voltage HV instrumentation wires in ITER magnet coils [46] and in the FUSILLO curved CCT magnet [47].

**Figure 4:**
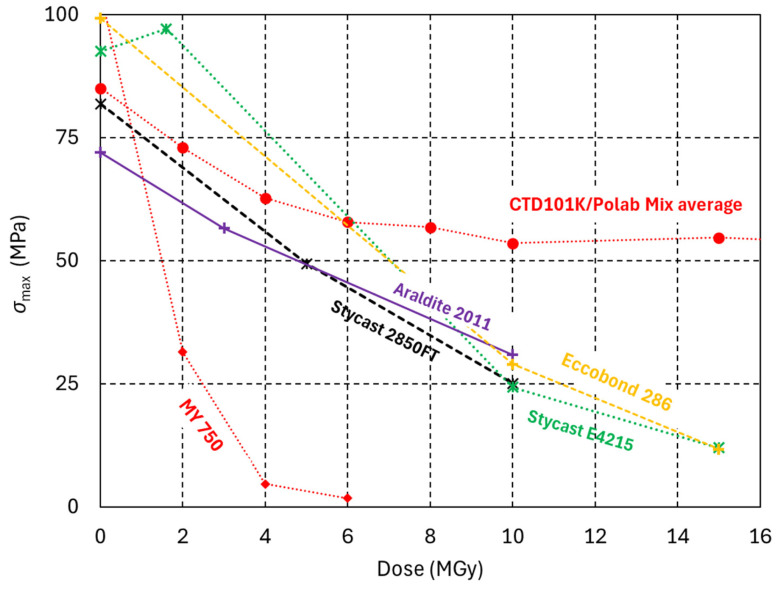
Maximum three-point bending stress (*σ*_max_) of epoxy adhesives as a function of radiation dose. The amine-based MY750 and the anhydride-based CTD101K/PolabMix epoxy systems are included for comparison.

The dose effect on the mechanical properties of the four epoxy systems with amine hardeners is similar (Figure 4). Their mechanical strength is halved, and *σ*_max_ is reduced below 50 MPa after a dose of about 5 MGy. Both Stycast composites exhibit strong irradiation-induced swelling at doses >5 MGy, and they are unusable after 10 MGy.

### 3.4. High-Performance and Engineering Polymers

**Figure 5:**
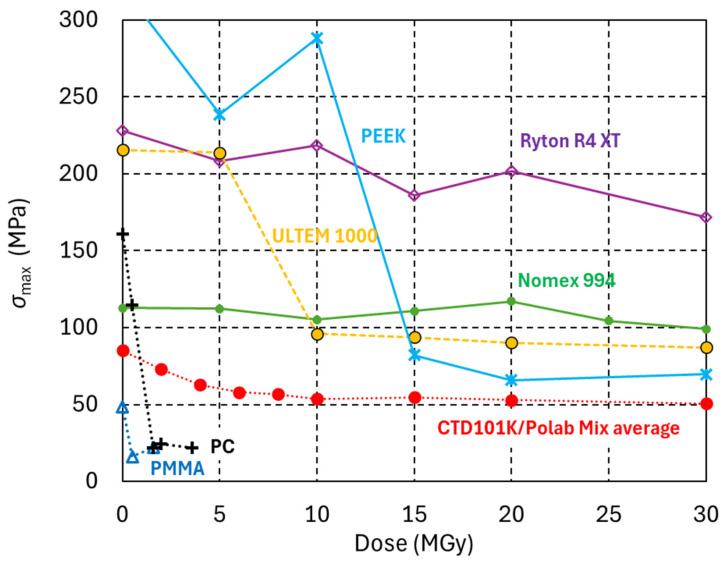
Maximum three-point bending stress (*σ*_max_) of different high-performance polymers as a function of radiation dose. The data for the widely used engineering plastic Polycarbonate (PC), of Poly (methyl methacrylate) (PMMA) and of an anhydride-based epoxy resin system is added for comparison.

Figure 5 compares the dose-dependent *σ*_max_ evolution of four high-performance polymers.

- Ryton R4 XT is thermoplastic composite composed of polyphenylene sulfide (PPS) resin reinforced with 40% glass fibres.
- ULTEM 1000 is a pure polyetherimide (PEI) high-temperature amorphous thermoplastic.

Ryton R4 XT and ULTEM 1000 are used in LHC interconnects [48] and in HL-LHC magnets. Test samples have been produced by injection moulding.

- PEEK (Polyetheretherketon) semicrystalline thermoplastic.
- Nomex 994 high-density, rigid pressboard made from Nomex aramid fibres is used, for instance, for resistive quadrupole magnets of the PCB booster.

Nomex 994 and Ryton R4 XT maintain most of their mechanical strength up to at least 30 MGy. PEEK has outstanding mechanical properties before irradiation and up to 10 MGy. After 15 MGy, its mechanical strength is strongly reduced, and it remains constant with higher doses up to at least 30 MGy. ULTEM 1000 shows a similar trend, with a strong reduction in mechanical strength after 10 MGy and then nearly constant *σ*_max_ up to at least 30 MGy.

### 3.5. Dose-Dependent Bending Strength of 3D-Printed Materials

The radiation hardness of five FDM-printed high-performance thermoplastics, notably PEEK, Helios PEEK, PEKK-A, the PEI-PC blend ULTEM 9085 [49], and the pure PEI ULTEM 1100, was compared with that of the SLA-printed Accura 25 and RG35 and with that of the SLS printed nylon PA2200. More information about pure PEEK, charged PEEK (Helios PEEK), ULTEM 9085 and the UV-curable resins Accura 25 and RG35 can be found in reference [50]. Polyamide 12 (EOS trade name PA2200) powder is commonly used for SLS printing.

**Figure 6:**
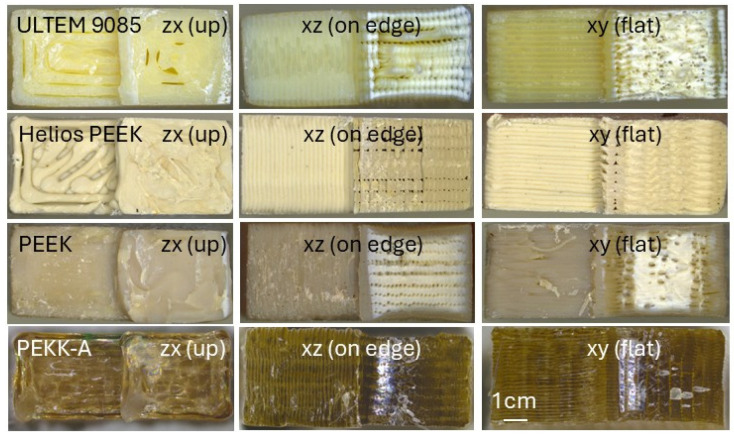
Fracture cross-sections of FDM-printed ULTEM 9085, Helios PEEK, PEEK and PEKK-A built in zx, xy and xz directions.

FDM-printed polymers exhibit anisotropic material properties. In the z-direction, the mechanical strength depends on the adhesive strength between the layers, which is weaker than the strength of the corresponding bulk material. Therefore, when loaded in the upright (zx) direction, the samples are weaker than in the xz and xy directions. The mechanical properties and anisotropy of the FDM-printed parts are influenced by the printing parameters and printing equipment [51]. The fracture surfaces of the FDM-printed thermoplastics, shown in Figure 6, reveal the filament roads and microscopic gaps between the filaments.

**Figure 7:**
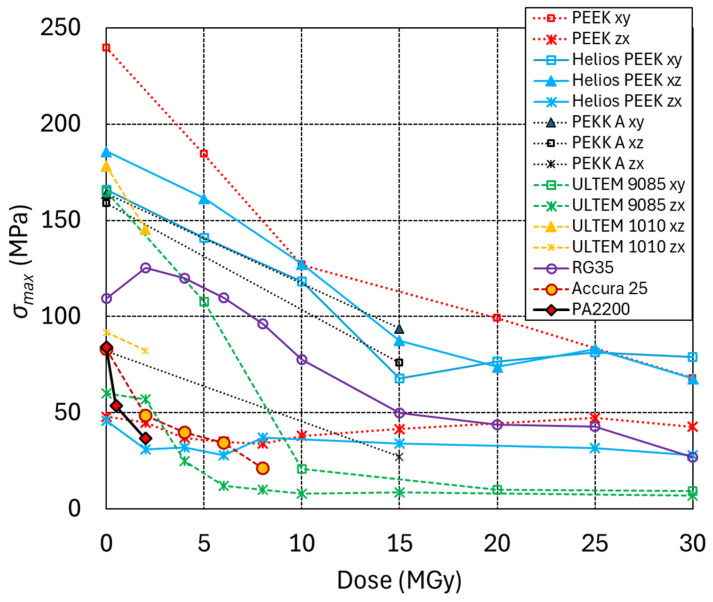
Maximum three-point bending stress (*σ*_max_) of 3D-printed polymers as a function of radiation dose.

In Figure 7, the maximum three-point bending stress (*σ*_max_) of the 3D-printed materials is plotted as a function of radiation dose. PEEK, Helios PEEK and PEKK-A exhibit comparatively good radiation hardness. After a dose of 10 MGy, their mechanical strength is reduced by about 50%. After a dose of 15 MGy, a plateau is reached, and substantial mechanical strength is maintained up to at least 30 MGy. The mechanical strength of PEEK and Helios PEEK in the upright printing direction is only moderately affected by doses up to at least 30 MGy. The tested 0 MGy PEKK A samples exhibit less mechanical anisotropy then the PEEK and Helios PEEK samples. However, after irradiation, a comparatively stronger reduction in strength in the zx direction is observed.

The mechanical strength of ULTEM 9085 is halved after an absorbed dose of about 5 MGy, and after 10 MGy, ULTEM 9085 loses most of its mechanical strength. ULTEM 9085 printed in the upright direction is already strongly degraded after 5 MGy.

FDM-printed ULTEM 1010 has so far only been irradiated up to 2 MGy, with a reduction in mechanical strength of about 20%.

The mechanical strength of SLA-printed RG35 is halved after a dose of about 15 MGy, and it maintains some mechanical strength up to 30 MGy. The mechanical strength of SLA Accura 25 is halved after a dose of about 5 MGy.

The least radiation-hard 3D material in the present study is the SLS-printed PA2200, with a 60% reduction in mechanical strength after a dose of 2 MGy.

### 3.6. Irradiation-Induced Changes in Shore A Hardness and Tensile Strain at Fracture of Polyurethane Elastomers

**Figure 8:**
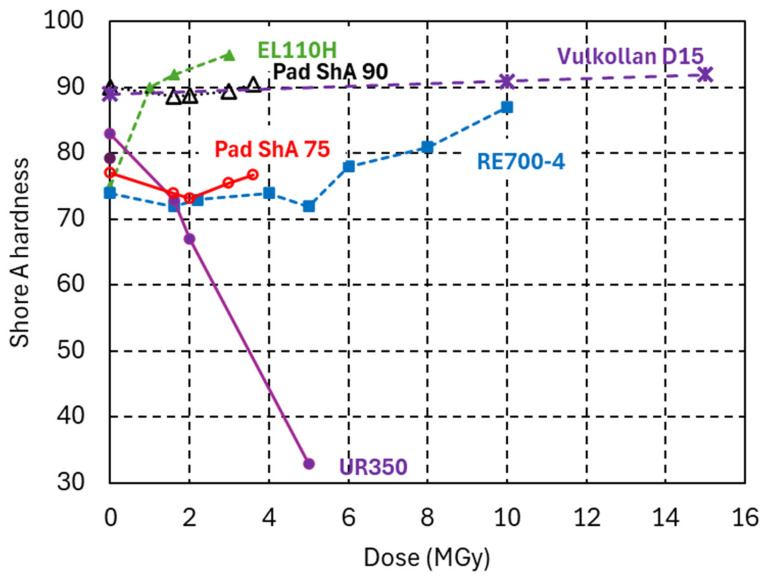
Shore A hardness of PUR elastomers Vulkollan D15, Pad ShA 75 and Pad ShA 90 as a function of the dose absorbed in ambient air. PUR EL110H, RE700-4 and UR350 from [52] are shown for comparison.

In Figure 8, the Shore A hardness evolution of the PUR systems is plotted as a function of the gamma dose absorbed in ambient air. Irradiation-induced cross-linking causes a ShA increase in the EL110H system. The initial hardness of the RE700-4 system of 74 ± 1 Shore A 3 s remains nearly constant up to a dose of 5 MGy, suggesting similar irradiation-induced cross-linking and chain scission rates. Further increasing the dose to 10 MGy increases the hardness to 87 ± 0.6 Shore A 3 s. The PUR system UR350 is chain-scission-dominated, as seen by the decrease in ShA hardness, *G’*_rubbery_, and *E*_RT_ [52]. The PUR Vulkollan D15 has outstanding radiation hardness, manifested by a nearly constant ShA hardness up to 15 MGy.

**Figure 9:**
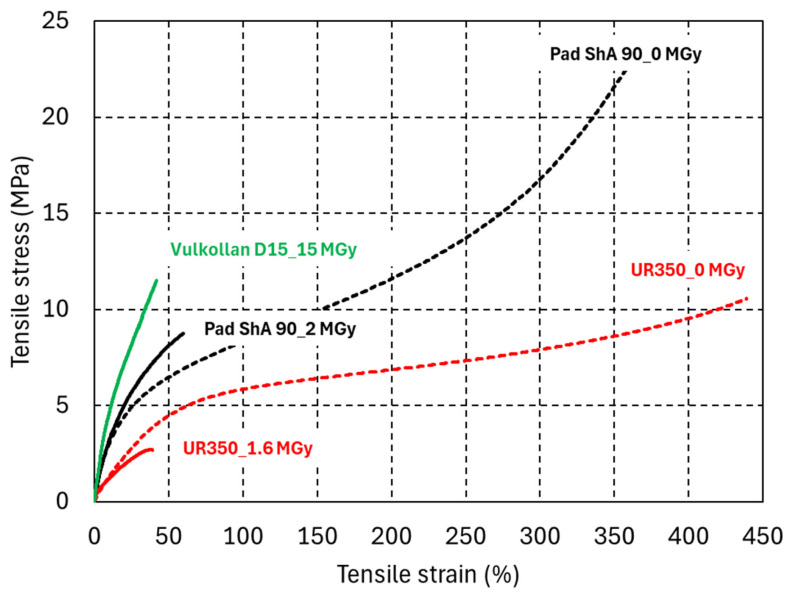
Uniaxial tensile engineering stress–strain curves of PUR elastomers Pad ShA 90,UR350, and Vulkollan D15 before and after irradiation.

Figure 9 compares the tensile stress–strain curves of PUR elastomers before and after irradiation. The tensile strain at fracture is more sensitive to radiation damage than the corresponding ShA hardness changes. After a dose of 15 MGy, the PUR Vulkollan D15 retains a tensile strain at fracture of 40%. A dose ten times lower is sufficient to reduce the strain at fracture of UR350 to the same level.

### 3.7. Irradiation-Induced Degradation of Breakdown Voltage

**Figure 10:**
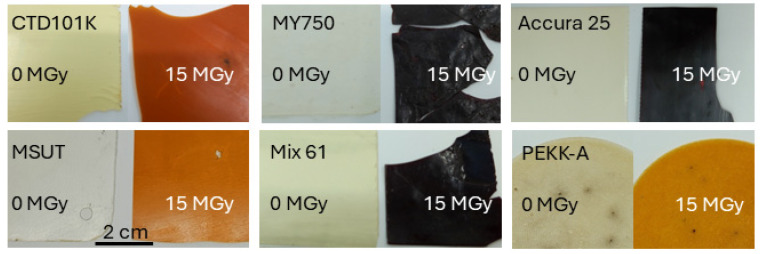
Samples irradiated with 0 MGy and 15 MGy after breakdown voltage test in liquid nitrogen.

The samples used for breakdown voltage tests, about 0.5 mm thick, are presented in Figure 10. After a dose of 15 MGy, the amine-based epoxy systems turned black and could not withstand significant mechanical stress without fracturing. 

**Figure 11:**
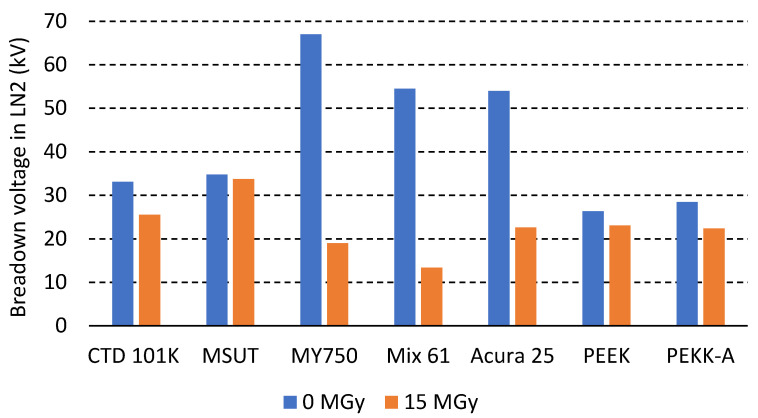
Breakdown voltage measured in LN2 before and after 15 MGy irradiation.

Figure 11 compares the breakdown voltage in LN2 of two anhydride-based epoxy systems (CTD101K and MSUT), three amine-based epoxy systems (MY750, Mix 61 and Accura 25) and FDM-printed PEEK and PEKK-A before and after 15 MGy irradiation.

After a dose of 15 MGy, the breakdown voltage of the amine-based systems in LN2 is reduced by about 70%. After the same dose, the breakdown voltage of the anhydride-based epoxy systems, as well as that of PEEK and PEKK-A, is reduced by only about 15%.

When the tested samples are not dense, the measured breakdown voltage is influenced by the polymer material and by the breakdown strength of LN2. For a gap distance of 0.5 mm, corresponding with the sample thickness, the breakdown voltage reported for LN2 is roughly 30 kV [53].

## 4. Discussion and Conclusions

In the present study, a wide range of polymeric materials was irradiated in ambient air with an approximate dose rate of 2 kGy/h up to 30 MGy. The good dose resolution of the experiment, between 0.5 and 5 MGy, enabled us to monitor the evolution of radiation damage in better detail than what was possible in previous irradiation damage studies.

Two groups of materials can be distinguished from the dose-dependent mechanical test results. For some polymers (including the anhydride-based epoxy systems, PEEK and PEI ULTEM 1000), after an initial decrease in mechanical properties, a stress plateau is reached, and it remains nearly unchanged up to at least 30 MGy (the maximum dose achieved so far in the present study). For other polymers (including the amine-based epoxy systems), such a plateau is not observed, and mechanical properties degrade with increasing dose until the material is unusable.

Typically, the radiation dose limit of a polymer is defined as the dose at which a critical material property decreases by a specified fraction, for instance, when the stress at fracture or the strain at fracture are halved. However, for materials exhibiting a stress plateau, such criteria may substantially underestimate their potential to resist high radiation doses.

Therefore, in Table 1, we compare the radiation hardness results derived from three-point bending strength with a radiation hardness ranking that has been established using two criteria: (i) the dose at which the initial bending strength is reduced by 50% and (ii) the dose at which the bending strength is reduced below 50 MPa. The 50 MPa threshold value was arbitrarily chosen to distinguish materials that retain substantial mechanical strength after high-dose irradiation. For materials that maintain bending strengths above 50 MPa after 30 MGy, irradiation to higher doses is ongoing to derive their useful dose limit.

The meta-aramid fibre pressboard (Nomex 994) and the charged PPS (Ryton R4 XT) have outstanding radiation hardness with only moderate changes in mechanical strength up to at least 30 MGy. The mechanical strength of PEEK, PEI ULTEM 1000, and of all epoxy resin systems with pure anhydride hardeners is halved after a dose in the range of 5 to 15 MGy. After the initial decrease, their mechanical strength remains nearly constant up to at least 30 MGy. All epoxy systems with amine-based hardeners are strongly degraded after a dose of 5 MGy.

The FDM 3D-printed PEEK, Helios PEEK and PEKK-A have similarly good radiation hardness as the machined PEEK. The FDM-printed materials exhibit strongly anisotropic mechanical properties, with much lower strength in the upright printing direction as machined PEEK samples. The FDM-printed PEI-PC blend ULTEM 9085 is less radiation-hard than the machined pure PEI ULTEM 1000. Irradiation can further reduce mechanical strength in the upright printing direction and increase mechanical anisotropy. This needs to be considered when using FDM-printed parts as functional components in an irradiation environment. Among the SLA-printed thermosets, RG35 exhibits outstanding radiation hardness. SLS-printed PA12 nylon (PA2200) is the least radiation-hard 3D-printed material in the present study.

Irradiation-induced changes in dielectric properties were assessed by breakdown voltage measurements in LN2. After exposure to a dose of 15 MGy, even the most degraded amine-based epoxy systems retained high breakdown voltages. The mechanical properties of the amine-based epoxy systems were already dramatically degraded after doses much lower than 15 MGy, indicating that the degradation of mechanical properties precedes the substantial degradation of their breakdown strength in dielectric liquids.

To precisely determine the dose limits of organic materials in superconducting magnets, the effects of the presence of oxygen and of irradiation temperature [33] need to be considered and will be the subject of further studies.

## Figures and Tables

**Table 1:** Radiation hardness ranking based on the dose at which *σ*_max_ is reduced by 50% (Dose *σ*_max_ = 50%) and the dose where *σ*_max_ is reduced below 50 MPa (Dose *σ*_max_ < 50 MPa).

Material	Dose *σ*_max_ = 50% (MGy)	Dose *σ*_max_ < 50 MPa (MGy)
Nomex 994_meta-aramid	>30	>30
Ryton R4 XT_polyphenylene sulfide (PPS)	>30	>30
PEEK_polyether ether ketone	10–15	>30
CTD425_epoxy-cyanate ester blend	10–15	>30
ULTEM 1000_polyetherimide (PEI)	5–10	>30
POLAB Mix_epoxy+anhydride hardener+DY040 flexibiliser	~10	>30
CTD101K_epoxy+anhydride hardener	~10	>30
MSUT (MY740 epoxy+HY 906 anhydride hardener +DY 062 accelerator)	~10	>30
Damisol 3418_epoxy+anhydride hardener	~15	>30
Araldite F epoxy+HY906 anhydride hardener blend+DY 040 flexibiliser	10–15	10–15
Mix 61 epoxy + amine hardener	~10	~5
MY750 epoxy + HY5922 amine hardener	~2	~2
Araldite 2011_epoxy+amine hardener	~5	~5
Eccobond 286 ^1^_epoxy+amine hardener	~5	~5
Stycast 2850FT+CAT23LV	~5	~5
Stycast E4215+HD3561	~5	~5
RG35 SLA	~10	15
Accura 25 SLA	2	4
Helios PEEK FDM flat and on edge	~10	>30
Helios PEEK FDM upright	>30	below 50 MPa at 0 MGy
PEEK FDM flat and on edge	~10	>30
PEEK FDM upright	>30	below 50 MPa at 0 MGy
PEKK A FDM flat and on edge	~10	>30
PEKK A FDM upright	~10	~5
ULTEM 9085 FDM flat and on edge_PEI-PC blend	~7	~8
ULTEM 9085 FDM upright	~3	~2
PA2200_PA12 nylon	~1	~0.5
PC Polycarbonate	~1	~1
PMMA Poly (methyl methacrylate)	<0.5	below 50 MPa at 0 MGy

^1^ Also known as Loctite Ablestik 286.

## Data Availability

The original contributions presented in this study are included in the article. Further inquiries can be directed to the corresponding author.
